# Interactions of Habitual Coffee Consumption by Genetic Polymorphisms with the Risk of Prediabetes and Type 2 Diabetes Combined

**DOI:** 10.3390/nu12082228

**Published:** 2020-07-26

**Authors:** Taiyue Jin, Jiyoung Youn, An Na Kim, Moonil Kang, Kyunga Kim, Joohon Sung, Jung Eun Lee

**Affiliations:** 1 Department of Food and Nutrition, College of Human Ecology, Seoul National University, Seoul 08826, Korea; taewol@snu.ac.kr (T.J.); ji0youn@snu.ac.kr (J.Y.); ank1101@snu.ac.kr (A.N.K.); 2 Institute of Health and Environment, Graduate School of Public Health, Seoul National University, Seoul 08826, Korea; kmihoho1@snu.ac.kr; 3 Statistics and Data Center, Research Institute for Future Medicine, Samsung Medical Center, Seoul 03181, Korea; kyunga.j.kim@samsung.com; 4 Department of Digital Health, Samsung Advanced Institute for Health Sciences & Technology, Sungkyunkwan University, Seoul 06351, Korea; 5 Department of Epidemiology, Graduate School of Public Health, Seoul National University, Seoul 08826, Korea; jsung@snu.ac.kr; 6 The Research Institute of Human Ecology, Seoul National University, Seoul 08826, Korea

**Keywords:** coffee consumption, type 2 diabetes, prediabetes, genome-wide association analysis (GWAS), single nucleotide polymorphism (SNP)

## Abstract

Habitual coffee consumption and its association with health outcomes may be modified by genetic variation. Adults aged 40 to 69 years who participated in the Korea Association Resource (KARE) study were included in this study. We conducted a genome-wide association study (GWAS) on coffee consumption in 7868 Korean adults, and examined whether the association between coffee consumption and the risk of prediabetes and type 2 diabetes combined was modified by the genetic variations in 4054 adults. In the GWAS for coffee consumption, a total of five single nucleotide polymorphisms (SNPs) located in 12q24.11-13 (rs2074356, rs11066015, rs12229654, rs11065828, and rs79105258) were selected and used to calculate weighted genetic risk scores. Individuals who had a larger number of minor alleles for these five SNPs had higher genetic risk scores. Multivariate logistic regression models were used to estimate the odds ratios (ORs) and 95% confidence intervals (95% CIs) to examine the association. During the 12 years of follow-up, a total of 2468 (60.9%) and 480 (11.8%) participants were diagnosed as prediabetes or type 2 diabetes, respectively. Compared with non-black-coffee consumers, the OR (95% CI) for ≥2 cups/day by black-coffee consumers was 0.61 (0.38–0.95; *p* for trend = 0.023). Similarly, sugared coffee showed an inverse association. We found a potential interaction by the genetic variations related to black-coffee consumption, suggesting a stronger association among individuals with higher genetic risk scores compared to those with lower scores; the ORs (95% CIs) were 0.36 (0.15–0.88) for individuals with 5 to 10 points and 0.87 (0.46–1.66) for those with 0 points. Our study suggests that habitual coffee consumption was related to genetic polymorphisms and modified the risk of prediabetes and type 2 diabetes combined in a sample of the Korean population. The mechanisms between coffee-related genetic variation and the risk of prediabetes and type 2 diabetes combined warrant further investigation.

## 1. Introduction

The incidence rate and prevalence of type 2 diabetes have steadily increased in Asian populations. The International Diabetes Federation (IDF) estimated that 163 million people (35.2% of global diabetic population) in the Western Pacific region had prevalent type 2 diabetes in 2019 [1], contributing the most to type 2 diabetes in the world. In Korea, the prevalence of type 2 diabetes has increased from 6.9% in 1998 to 10.8% in 2017 [2,3]. Additionally, type 2 diabetes contributed to 17.1% of the total deaths in Korea in 2018 [4].

Coffee consumption has been suggested to lower several chronic diseases, including type 2 diabetes [5], metabolic syndrome [6], coronary heart disease [7], liver disorders [8], and several types of cancers [9]. The bioactive compounds in coffee, such as caffeine and chlorogenic acids, have been investigated as potential compounds that lower the risk of type 2 diabetes. Caffeine has been shown to stimulate the metabolic rate [10,11], and its thermogenic effect has been hypothesized to decrease the risk of metabolic disease development. Antioxidants, including chlorogenic acids, commonly found in coffee, have also been highlighted as a preventing factor for type 2 diabetes by inhibiting the generation of free radicals and removing hyperglycemia-induced oxidative stress [12,13,14].

A heritability study on caffeine [15] and genome-wide association studies (GWASs) have suggested that coffee consumption behavior may be linked to genetic polymorphisms. The first genome-wide meta-analysis of coffee consumption was conducted in a European population and identified two independent loci, rs4410790 nears *AHR* and rs2470893 between *CYP1A1* and *CYP1A2*, which are caffeine metabolism-related genes [16]. Additional European/Caucasian GWASs also discovered single nucleotide polymorphisms (SNPs) located in *AHR*, *CYP1A1* and *CYP1A2* as well as *ABCG2*, *POR*, *BDNF*, *SLC6A4*, *GCKR* and *MLXIPL* [17], near *NRCAM* or *ULK3* [18]. A Japanese GWAS, the first GWAS of coffee consumption in Asia, identified 24 SNPs on chromosome 12, showing rs2074356 in *HECTD4* as the strongest significant SNP [19]. Another Japanese GWAS found two loci located in *CUX2* (rs7910258) and *AHR* (rs10251701) [20]. The few GWASs in Asia warrant further investigation of the coffee-related genetic polymorphisms in Asian populations because there has been an increase in coffee consumption and type 2 diabetes in Asia. Because coffee consumption has the potential to prevent type 2 diabetes [21], it is important to investigate whether coffee consumption is linked to a lower risk of type 2 diabetes and whether this association is modified by genetic variations common in Asian populations.

The objective of this study was to identify genetic polymorphisms associated with habitual coffee consumption and examine whether the association between coffee consumption and the risk of prediabetes and type 2 diabetes combined was modified by these genetic variants in Korean population.

## 2. Materials and Methods

### 2.1. Study Population

Participants were recruited from the Korea Association Resource (KARE) study, which is part of the Korean Genome and Epidemiology Study (KoGES), a community-based cohort study. A total of 5012 and 5018 participants aged 40–69 years were enrolled from Ansan and Ansung, respectively, between 2001 and 2002. Socio-demographic status, anthropometric indices, dietary lifestyle, physical activity, and disease history were examined at baseline and follow-up phase every 2 years. In this study, we included data from baseline to the 6th follow-up (2001–2014). The details of the KoGES project have been described elsewhere [22].

Among the 10,030 participants of this study, DNA samples from a total of 8840 participants were genotyped at baseline. We excluded participants who did not have genetic information (*n* = 1190); those who were diagnosed with a type 2 diabetes by physicians or had been treated with oral hypoglycemic medication or insulin therapy at baseline (*n* = 619); those who had a history of cardiovascular disease including myocardial infarction and stroke (*n* = 150) or cancer (*n* = 20) at baseline; and those who did not provide daily coffee consumption frequency (*n* = 129) or amount (*n* = 54). In summary, 7868 participants (3685 men and 4183 women) were included in the GWAS (Table S1).

When we examined the association between habitual coffee consumption and the incident risk of prediabetes and type 2 diabetes combined, we further excluded participants who had prevalent type 2 diabetes (*n* = 666) or prediabetes (*n* = 2099) determined by the fasting plasma glucose (FPG) test, a 2-h oral glucose tolerance test (OGTT) and a hemoglobin A1c (HbA1c) test at baseline. We also excluded those who attended neither fifth nor sixth follow-up examination (*n* = 1049). As a result, a total of 4054 participants (1904 men and 2150 women) were included in the association analysis. This study was approved by the Institutional Review Board of Seoul National University (IRB No. E1911/003-011).

### 2.2. Dietary Assessment

A 103-item semi-quantitative food frequency questionnaire (FFQ) for the KoGES was used to assess the habitual coffee consumption at baseline. The validity of the FFQ was evaluated by 3-day diet records [23,24]. Participants were asked to answer the frequency of coffee consumption over the last year (almost none, 1 time/month, 2–3 times/month, 1–2 times/week, 3–4 times/week, 5–6 times/week, 1 time/day, 2 times/day or 3 times/day) as well as the amount of coffee (0.5, 1 or 2 cups per time). The amount of sugar added in coffee was also investigated (1, 2 or 3 teaspoons per time). We multiplied the frequency and the amount of the daily consumption of coffee to obtain the number of cups consumed per day. Coffee consumption was categorized into non-coffee consumers, <1 cup/day, 1 to <2 cups/day and ≥2 cups/day. Among coffee consumers, participants who consumed coffee without sugar were defined as black-coffee consumers and those who consumed coffee with sugar as sugared-coffee consumers.

We calculated the amount of caffeine consumption of each participant by using the caffeine database published by the Korea Ministry of Food and Drug Safety (MFDS) [25] or the United States Department of Agriculture (USDA) [26] or by contacting the product companies. The foods considered to contain caffeine were as follows: beverages (coffee, tea and soft drinks) and foods made with cocoa (chocolate, chocolate candies, chocolate ice cream, chocolate milk, chocolate pies, and chocolate cakes).

### 2.3. Ascertainment of Cases

Participants underwent a 2-h 75 g OGTT and HbA1c test at baseline and each follow-up. The concentrations of FPG and 2-h plasma glucose were measured by the hexokinase method (ADVIA 1650; Bayer, Berkley, MI, USA). Incident cases of prediabetes and type 2 diabetes were defined according to the American Diabetes Association (ADA) criteria [27]. Incident type 2 diabetes was ascertained if a participant had one of the following during the follow-up examination: had been diagnosed with a type 2 diabetes by physicians or had been treated with oral hypoglycemic medication or insulin therapy; had an FPG ≥ 126 mg/dL (≥7.0 mmol/L); had a 2-h plasma glucose ≥ 200 mg/dL (≥11.1 mmol/L); or had a HbA1c ≥ 6.5%. Prediabetes incidence was defined as an FPG ≥ 100 mg/dL (≥5.6 mmol/L) or 2-h plasma glucose ≥ 140 mg/dL (≥7.8 mmol/L) or HbA1c ≥ 6.0%.

### 2.4. Genotyping and Quality Control

DNA samples were genotyped using the Affymetrix Genome-Wide Human SNP Array 5.0 (Affymetrix, Santa Clara, CA, USA). The Bayesian Robust Linear Modeling using Mahalanobis Distance (BRLMM) Genotyping Algorithm was used for the genotype calling of 500,568 SNPs [28]. After quality control filtering, a total of 352,228 SNPs remained for analysis. Details about quality control criteria have been described elsewhere [29].

### 2.5. GWAS on Coffee Consumption

We identified SNPs related to habitual coffee consumption in the GWAS. Participants were grouped into non-coffee consumers and coffee consumers, and this group information was used as a binary outcome variable in a logistic regression model for each SNP. We adjusted for age (years, continuous), sex, and alcohol consumption (g/day, continuous) in the GWAS. We also adjusted for baseline BMI in the secondary GWAS. In addition, we replicated a GWAS using a continuous variable of coffee consumption. A GWAS on continuous caffeine intake was also conducted. A box-cox transformed coffee or caffeine intake and a linear regression model were used for continuous analysis. A Manhattan plot and quantile-quantile (Q-Q) plot were generated, and the inflation factor (λ) was calculated. The GWAS was performed using PLINK version 1.07, and SNPs with a *p*-value < 1 × 10^−5^ were considered suggestive significant. A regional association plot of the significant SNPs was generated using the LocusZoom program (http://locuszoom.org). The linkage disequilibrium (LD) between the significant SNPs was examined using the HaploView program [30].

### 2.6. Statistical Analysis

To calculate the genetic risk scores, each coffee-related SNP identified from the GWAS was assigned 0, 1, or 2 according to the number of minor alleles and then weighted by its relative effective size (β coefficient obtained from the GWAS). The genetic risk scores were calculated as following the equation: 5 × (β_1_ × SNP_1_ + β_2_ × SNP_2_ + β_3_ × SNP_3_ + β_4_ × SNP_4_ + β_5_ × SNP_5_)/(β_1_ + β_2_ + β_3_ + β_4_ + β_5_) [31]. The genetic risk scores ranged from 0 to 10 points, and the median value was 5 points among those with at least one minor allele. Participants were categorized into 3 groups: 0 point, 0.1 to <5 points, and 5 to 10 points. We examined the association between coffee consumption and the risk of prediabetes and type 2 diabetes combined in black coffee and sugared coffee. Non-coffee consumers and sugared-coffee consumers combined were regarded as reference group for black-coffee consumers. For sugared-coffee consumers, non-coffee consumers and black-coffee consumers combined were regarded as a reference group. Odds ratios (ORs) and 95% confidence intervals (CIs) for the association between coffee consumption and the risk of prediabetes and type 2 diabetes combined were calculated using multivariate logistic regression models among men and women combined or separately. The median cups of coffee consumed per day were assigned to each coffee group and used to test the linear trends. We also examined whether the association varied by genetic risk scores. The interaction analysis was performed by comparing the models with or without interaction term using a likelihood ratio test. All of the analyses were adjusted for age (years, continuous), sex (for men and women combined), body mass index (BMI, <23, 23 to <25, 25 to <30 and ≥30 kg/m^2^), smoking status (never smokers, ≤10 and >10 pack-years for black-coffee consumers; never smokers, ≤10, 10.1 to ≤20, 20.1 to ≤30 and >30 pack-years for sugared-coffee consumers), alcohol consumption (non-drinkers, ≤5, 5.1 to ≤10 and >10 g/day for black-coffee consumers; non-drinkers, ≤5, 5.1 to ≤10, 10.1 to ≤20 and >20 g/day for sugared-coffee consumers), family history of type 2 diabetes (yes or no), and total energy intake (kcal/day, continuous). Additionally, we also adjusted for the amount of sugar added in coffee when analyzing the association between black coffee consumption and the risk of prediabetes and type 2 diabetes combined (0, ≤5, 5.1 to ≤10, 10.1 to ≤15 and >15 g/day). SAS version 9.4 (SAS Institute, Cary, NC, USA) was used for all analyses, and *p*-value < 0.05 in two-sided tests was defined as a significant difference.

## 3. Results

### 3.1. Baseline Characteristics

Of the 4054 participants in the association analysis, a total of 480 (11.8%) and 2468 (60.9%) participants developed type 2 diabetes and prediabetes during the 12-year follow-up period, respectively. Table 1 shows the baseline characteristics of the study population according to habitual coffee consumption. Compared with non-coffee consumers, participants who consumed either black coffee or sugared coffee were more likely to be younger, smoke, drink alcohol, and have a higher BMI.

### 3.2. Genetic Polymorphisms Associated with Coffee Consumption

In the GWAS of habitual coffee consumption, a total of 18 SNPs located in 12q24 achieved a suggestive significance (*p* < 1 × 10^−5^) (Table S2). When we additionally adjusted for baseline BMI, the same 18 significant SNPs were identified as well (Table S3). SNPs identified based on habitual coffee consumption were the same when we additionally conducted a GWAS for continuous caffeine intake.

**Figure 1:**
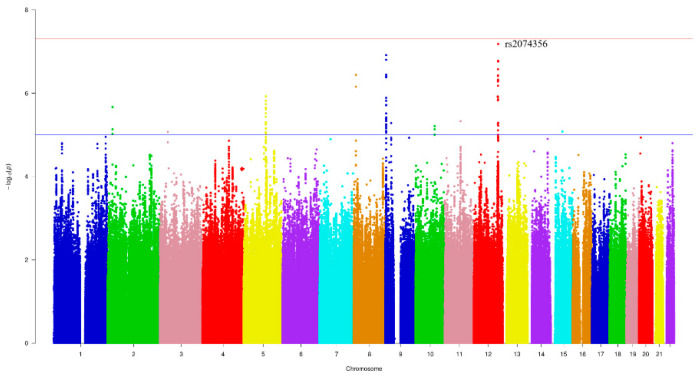
Manhattan plot of the genome-wide association study (GWAS) on coffee consumption. Each color represents a different chromosome. The strongest significant SNP was rs2074356 in chromosome 12 (*p*-value = 6.62 × 10^−8^).

**Figure 2:**
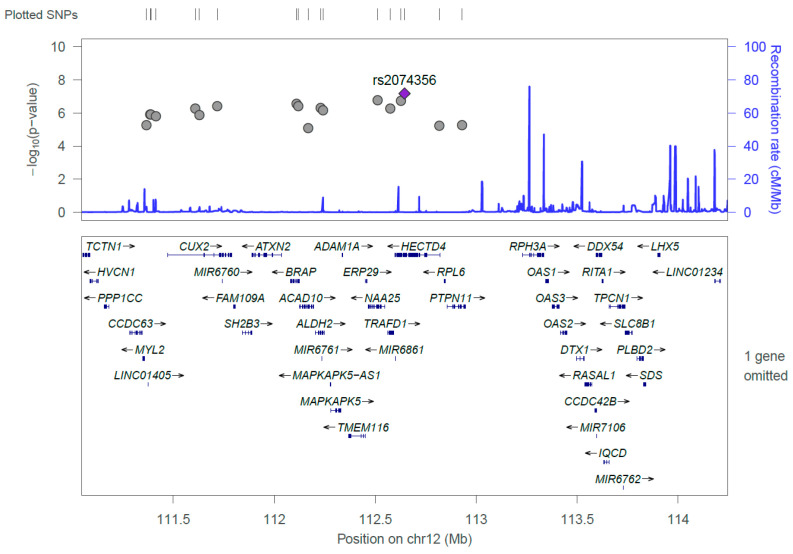
Regional association plot of the 18 significant single nucleotide polymorphisms (SNPs) discovered from the GWAS on coffee consumption. The strongest significant SNP, rs2074356, was shown in purple, and the gray dots represent chromosomal positions of other SNPs near the rs2074356.

**Figure 3:**
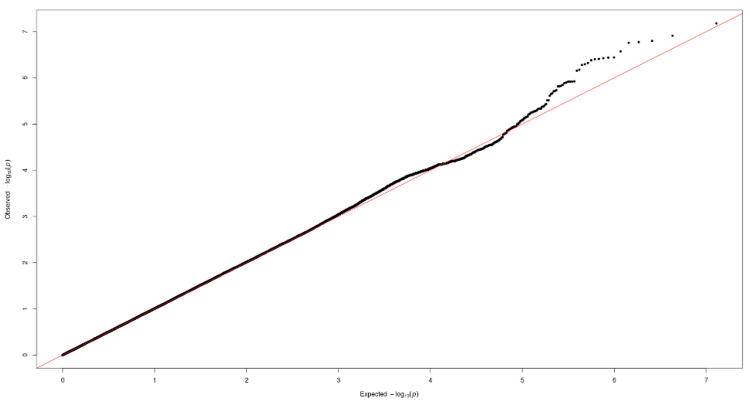
Quantile-quantile (Q-Q) plot of the GWAS on coffee consumption.

**Figure 4:**
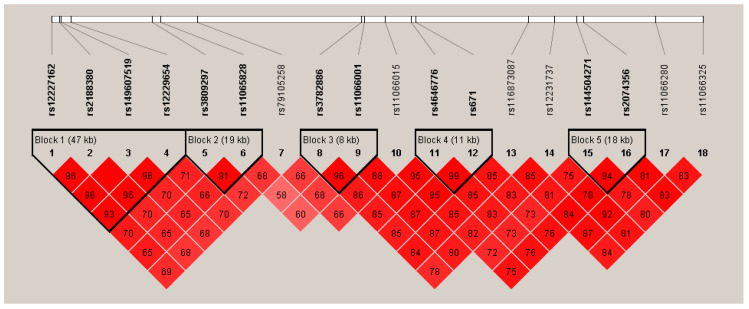
Linkage disequilibrium (LD) plot of the 18 significant SNPs discovered from the GWAS on coffee consumption. Values shown in the red boxes represent the LD (r^2^) between the SNPs.

Figure 1 and Figure 2 show the Manhattan plot and regional association plot, respectively. Figure 3 shows the Q-Q plot, and the genomic inflation factor (λ) of the GWAS was 1.0103, suggesting that the study population structure was well-adjusted [32]. Among the SNPs achieved suggestive significance at *p* < 1 × 10^−5^, we selected SNPs as follows, which were used later for calculating the genetic risk scores. First, pairwise correlations were examined and high correlation was declared when r^2^ > 0.8 (Figure 4). Then, we excluded all imputed SNPs having a high correlation with any genotyped SNP, and selected the SNPs among highly-correlated genotyped SNPs. As a result, three genotyped SNPs (rs2074356, rs11066015, and rs12229654) and two imputed SNPs (rs11065828 and rs79105258) were considered as coffee-related SNPs and used for calculating the genetic risk scores. Table 2 presents the information on these five SNPs related to coffee consumption. The SNPs were located in genes *HECTD4*, *ACAD10*, *MYL2*, and *CUX2*, and the minor allele frequency ranged from 0.143 to 0.172 in our population.

### 3.3. Coffee Consumption and the Risk of Prediabetes and Type 2 Diabetes Combined

We examined the association between black coffee consumption and the risk of prediabetes and type 2 diabetes combined (Table 3). Compared with non-black-coffee consumers, participants who consumed ≥2 cups/day of black coffee had a 39% lower risk of prediabetes and type 2 diabetes combined among men and women combined (95% CI = 0.38–0.95; *p* for trend = 0.023). When we separated men and women, compared with non-black-coffee consumers, participants who consumed ≥2 cups/day of black coffee had a 54% lower risk of prediabetes and type 2 diabetes combined among men (95% CI = 0.23–0.94; *p* for trend = 0.026). Although the association was not statistically significant among women, an inverse trend was observed (OR = 0.74; 95% CI = 0.41–1.34).

Table 4 presents the association between sugared coffee consumption and the risk of prediabetes and type 2 diabetes combined. The ORs (95% CIs) of prediabetes and type 2 diabetes combined comparing sugared-coffee consumers with non-sugared-coffee consumers were 0.73 (0.60–0.89; *p* for trend = 0.005) for men and women combined, 0.71 (0.52–0.97; *p* for trend = 0.015) for men, and 0.75 (0.57–0.99; *p* for trend = 0.080) for women.

### 3.4. Associations Modified by Genetic Risk Scores

We analyzed whether the association between habitual coffee consumption and the risk of prediabetes and type 2 diabetes combined varied by genetic polymorphisms. For black-coffee consumers, we found that an inverse association was more pronounced among individuals with high genetic risk scores, but the interaction was not statistically significant (*p* for interaction = 0.261) (Table 5). The ORs (95% CIs) for ≥2 cups/day of black coffee vs. non-black-coffee consumers were 0.87 (0.46–1.66) for participants with 0 points, 0.49 (0.17–1.44) for those with 0.1 to <5 points, and 0.36 (0.15–0.88) for those with 5 to 10 points for their genetic risk scores. For sugared coffee consumption, the associations with the risk of prediabetes and type 2 diabetes combined were similar across genetic risk scores (*p* for interaction = 0.608) (Table 6). When we conducted an interaction analysis for each coffee-related SNP for black coffee consumption, the association between black coffee consumption and the risk of prediabetes and type 2 diabetes combined was inverse for the minor allele of each SNP (Table 7).

## 4. Discussion

Our first GWAS of coffee consumption in the Korean population identified five SNPs (rs2074356 in *HECTD4*, rs11066015 in *ACAD10*, rs12229654 in *MYL2*, rs11065828 and rs79105258 in *CUX2*) related to habitual coffee consumption. Compared with non-coffee consumers, the risk of prediabetes and type 2 diabetes being combined was inversely associated with habitual coffee consumption, either black coffee or sugared coffee. Individuals with black coffee consumption had a lower risk of prediabetes and type 2 diabetes combined compared with non-black-coffee consumers among those with multiple minor alleles for these five SNPs.

The significant SNPs discovered to be related to habitual coffee consumption in our GWAS were all introns. Although the introns were noncoding regions of genes, they may affect the transcription rate and translation efficiency, further regulating gene expression [33]. Recent GWASs have identified several loci on the *AHR*, *CYP1A1*, and *CYP1A2* genes associated with coffee consumption [16,17]. However, most of the GWASs on coffee consumption were conducted in European populations, only two GWASs, to our knowledge, reported SNPs related to coffee consumption in Asian populations. A GWAS in the Japan Multi-Institutional Collaborative Cohort (J-MICC) study, the first GWAS on coffee consumption in Asia, found that rs2074356 located in 12q24 was most strongly associated with habitual coffee consumption (*p* = 2.2 × 10^−6^) [19]. Similarly, in our GWAS, a total of 18 SNPs were associated with coffee consumption at *p* < 1 × 10^−5^, all of which were found to be related to habitual coffee consumption in the J-MICC study, and the strongest significant variant was rs2074356 as well. Another Japanese coffee GWAS identified two independent loci (rs79105258 in 12q24 and rs10252701 in 7p21) that were associated with coffee consumption [20]. rs79105258 was also selected as a coffee-related variant in our study. In addition to the association with habitual coffee consumption, these SNPs were associated with type 2 diabetes [34], blood glucose levels [35], blood pressure levels [36], and obesity [37]. rs2074356 in *HECTD4* was associated with prevalent type 2 diabetes and blood glucose level in a Korean population [34,35]. Three SNPs (rs12229654, rs11066015 and rs2074356) were also identified to be linked with both systolic and diastolic blood pressure in a Japanese GWAS [36].

Previous epidemiologic studies have shown that coffee consumption was inversely associated with the risk of type 2 diabetes. A meta-analysis of 28 cohort and nested case-control studies reported that participants who consumed 5 cups/day of coffee had a 30% lower risk of type 2 diabetes compared with almost non-consumers, and the associations were similar between men and women [5]. Although we found a stronger inverse association among men than among women, further investigation is needed to explore a larger amount of coffee consumption, e.g., 3 or more cups/day, and whether the inverse association holds for women.

The lower risk of type 2 diabetes linked to coffee consumption could be linked to several biological mechanisms. As a main polyphenolic compound in coffee, chlorogenic acids have been shown as inhibitors of hepatic glucose-6-phosphatase, the rate-limiting enzyme of glucose hydrolysis [38]. Reduced hepatic glucose-6-phosphatase may affect the glucose output and thus decrease the blood glucose concentration. In addition, chlorogenic acids act as antioxidants to lower oxidative stress shown in both in vitro and in vivo studies [39]. Additionally, caffeine and magnesium, both of which are commonly found in coffee, have been suggested to have roles in type 2 diabetes prevention by improving insulin resistance. Previous studies suggested that caffeine could improve insulin resistance by stimulating insulin secretion from pancreatic β cells [40]. In addition to insulin secretion, caffeine increases thermogenesis, lipolysis, and β-oxidation [41]. Magnesium supplementation has reduced the development of type 2 diabetes and improved glucose disposal in experimental studies [42,43], and cohort studies have reported a significant inverse association between magnesium intake and type 2 diabetes risk [44].

In this study, we observed that both black coffee and sugared coffee decreased the risk of prediabetes and type 2 diabetes combined for individuals who consumed more than 2 cups of coffee per day. But participants who consumed 1 to <2 cups/day of sugared coffee had a 45% higher risk of prediabetes and type 2 diabetes combined among men alone, but not among women. Although sugar-sweetened beverage intake including carbonated beverages has been positively associated with the risk of type 2 diabetes [45,46], the results of coffee with sugar remained equivocal. In a French cohort study, compared to non-coffee consumers, the incident type 2 diabetes decreased by 40% and 31% among participants consuming more than 1.1 cups of coffee per lunch with or without sugar, respectively [47]. In a small clinical trial, where eight lean, young and healthy adults drank six types of beverages or water 1 h before a potato-based meal, postprandial hyperglycemia, an early abnormality of type 2 diabetes [48], was significantly reduced when they drank sweetened coffee before their meals [49]. Further studies are needed to examine whether the benefit of coffee remains even after adding a small amount of sugar.

To our knowledge, this study is the first GWAS of habitual coffee consumption in a Korean population. The strengths of this study include good ascertainment of prediabetes and type 2 diabetes, adjustment for potential confounding factors, and a 12-year follow-up. The incidence of prediabetes and type 2 diabetes were identified based on the circulating levels of FPG, 2-h plasma glucose, and HbA1c, which could minimize the misclassification. Adjustment for potential confounding factors, including smoking status and alcohol consumption, may enable us to remove the effect of the confounding factors. However, we cannot rule out the possibility that residual confounding factors remained. There are several limitations to our study. First, the rate of revisits to the clinic for the blood draw decreased to 60% in the last sixth follow-up. Therefore, our study may not be representative of the full cohort of KARE study. However, the internal validity may not be impaired as we obtained relatively accurate information on the incidence of prediabetes and type 2 diabetes during the 12-year follow-up. Second, we could not distinguish caffeinated and decaffeinated coffee or boiled and filtered coffee. However, previous studies have shown that the associations between coffee and type 2 diabetes were similar by the amount of caffeine [5] or preparation methods [50]. Third, we were not able to examine high coffee consumption (e.g., 3 or more cups/day) because only a few participants consumed more than 3 cups/day. Fourth, we did not consider medicinal caffeine intake when we performed a GWAS. However, beverages may mainly contribute to daily caffeine intake in Korea.

## 5. Conclusions

In our study, we conducted a GWAS and discovered five SNPs (rs2074356 in *HECTD4*, rs11066015 in *ACAD10*, rs12229654 in *MYL2*, rs11065828 and rs79105258 in *CUX2*) associated with habitual coffee consumption in a Korean population. We observed that moderate black coffee and sugared coffee consumption reduced the risk of prediabetes and type 2 diabetes combined. We found that an inverse association was stronger among black-coffee consumers with minor alleles of five SNPs related to coffee consumption compared to those with major alleles. Further Asian GWASs and epidemiological studies are needed to elucidate the effects of coffee-related genetic variation and high coffee consumption on chronic disease risk.

## Figures and Tables

**Table 1:** Baseline characteristics of study population according to habitual coffee consumption.

	Non-Coffee Consumers (*n* = 864)	Black-Coffee Consumers		
		<1 Cup/Day (*n* = 100)	1 to <2 Cups/Day (*n* = 70)	≥2 Cups/Day (*n* = 105)
Age, mean ± SD (years)	53.3 ± 8.7	49.7 ± 7.5	47.4 ± 6.9	46.9 ± 5.7
Sex, *n* (%)				
Men	299 (34.6)	32 (32.0)	21 (30.0)	47 (44.8)
Women	565 (65.4)	68 (68.0)	49 (70.0)	58 (55.2)
BMI, mean ± SD (kg/m^2^)	23.8 ± 3.0	24.3 ± 2.8	24.6 ± 3.1	24.7 ± 3.1
Smoking status, *n* (%)				
Never smokers	638 (73.8)	76 (76.0)	49 (70.0)	57 (54.3)
Past smokers	103 (11.9)	9 (9.0)	11 (15.7)	14 (13.3)
Current smokers	123 (14.2)	15 (15.0)	10 (14.3)	34 (32.4)
Alcohol consumption, *n* (%)				
Never drinkers	588 (68.1)	55 (55.0)	44 (62.9)	46 (43.8)
≤5 g/day	104 (12.0)	15 (15.0)	12 (17.1)	23 (21.9)
5 to ≤10 g/day	36 (4.2)	8 (8.0)	4 (5.7)	8 (7.6)
10 to ≤20 g/day	46 (5.3)	10 (10.0)	6 (8.6)	11 (10.5)
>20 g/day	90 (10.4)	12 (12.0)	4 (5.7)	17 (16.2)
Family history of type 2 diabetes, *n* (%)				
Yes	65 (7.5)	11 (11.0)	5 (7.1)	14 (13.3)
No	799 (92.5)	89 (89.0)	65 (92.9)	91 (86.7)
Sugar added in coffee, mean ± SD (g/day)	0.1 ± 0.9	0 ± 0	0 ± 0	0 ± 0
Total energy intake, mean ± SD (kcal/day)	1882.1 ± 762.5	1981.1 ± 747.6	1863.9 ± 718.0	2143.7 ± 1121.4
	**Non-Coffee Consumers (*n* = 864)**	**Sugared-coffee Consumers**		
		**<1 Cup/Day (*n* = 892)**	**1 to <2 Cups/Day (*n* = 986)**	**≥2 Cups/Day (*n* = 1037)**
Age, mean ± SD (years)	53.3 ± 8.7	51.4 ± 8.5	50.0 ± 8.4	49.2 ± 8.0
Sex, *n* (%)				
Men	299 (34.6)	442 (49.6)	425 (43.1)	638 (61.5)
Women	565 (65.4)	450 (50.5)	561 (56.9)	399 (38.5)
BMI, mean ± SD (kg/m^2^)	23.8 ± 3.0	24.4 ± 2.9	24.3 ± 2.9	24.3 ± 3.0
Smoking status, *n* (%)				
Never smokers	638 (73.8)	555 (62.2)	626 (63.5)	458 (44.2)
Past smokers	103 (11.9)	147 (16.5)	129 (13.1)	188 (18.1)
Current smokers	123 (14.2)	190 (21.3)	231 (23.4)	391 (37.7)
Alcohol consumption, *n* (%)				
Never drinkers	588 (68.1)	435 (48.8)	488 (49.5)	459 (44.3)
≤5 g/day	104 (12.0)	166 (18.6)	196 (19.9)	204 (19.7)
5 to ≤10 g/day	36 (4.2)	77 (8.6)	59 (6.0)	85 (8.2)
10 to ≤20 g/day	46 (5.3)	90 (10.1)	88 (8.9)	91 (8.8)
>20 g/day	90 (10.4)	124 (13.9)	155 (15.7)	198 (19.1)
Family history of type 2 diabetes, *n* (%)				
Yes	65 (7.5)	104 (11.7)	91 (9.2)	96 (9.3)
No	799 (92.5)	788 (88.3)	895 (90.8)	941 (90.7)
Sugar added in coffee, mean ± SD (g/day)	0.1 ± 0.9	1.6 ± 1.5	4.9 ± 1.2	11.9 ± 4.4
Total energy intake, mean ± SD (kcal/day)	1882.1 ± 762.5	1881.2 ± 638.6	1969.5 ± 596.2	2115.8 ± 749.4

Abbreviations: SD, standard deviation; BMI, body mass index.

**Table 2:** SNPs related to coffee consumption (*p*-value < 1 × 10^−5^).

Chr	SNP	Position	Gene	Alleles ^1^	MAF ^2^	MAF ^3^	Beta ^4^	*p*-Value ^5^
12	rs2074356	112,645,401	*HECTD4*	A/G	0.149	0.129	0.3185	6.62 × 10^−8^
12	rs11066015	112,168,009	*ACAD10*	A/G	0.172	0.176	0.2469	7.79 × 10^−6^
12	rs12229654	111,414,461	*MYL2*	G/T	0.143	0.159	0.2867	1.49 × 10^−6^
12	rs11065828	111,629,389	*CUX2*	A/C	0.172	0.214	0.2654	1.26 × 10^−6^
12	rs79105258	111,718,231	*CUX2*	A/C	0.156	0.216	0.2912	3.89 × 10^−7^

Abbreviations: Chr, chromosome; SNP, single nucleotide polymorphism; MAF, minor allele frequency. ^1^ Alleles are presented as minor allele/major allele; ^2^ minor allele frequency in this study population; ^3^ minor allele frequency in the 1000Genomes, East Asian; ^4^ the beta (β) coefficient was obtained from the GWAS; ^5^ the *p*-value was calculated using a Wald test from logistic regression model adjusted for age (years; continuous), sex, and alcohol consumption (g/day; continuous).

**Table 3:** Multivariate-adjusted ORs and 95% CIs for the risk of prediabetes and type 2 diabetes combined according to black coffee consumption.

	Non-Black-Coffee Consumers ^1^	Black-Coffee Consumers			*p* for Trend
		<1 Cup/Day	1 to <2 Cups/Day	≥2 Cups/Day	
Men and women combined					0.023
Case/total	2759/3779	73/100	47/70	69/105	
ORs (95% CIs) ^2^	Reference	0.96 (0.59, 1.55)	0.75 (0.44, 1.28)	0.61 (0.38, 0.95)	
Men					0.026
Case/total	1394/1804	26/32	15/21	32/47	
ORs (95% CIs) ^2^	Reference	1.18 (0.45, 3.15)	0.69 (0.25, 1.93)	0.46 (0.23, 0.94)	
Women					0.271
Case/total	1365/1975	47/68	32/49	37/58	
ORs (95% CIs) ^2^	Reference	0.91 (0.52, 1.59)	0.78 (0.41, 1.47)	0.74 (0.41, 1.34)	

Abbreviations: OR, odds ratio; 95% CI, 95% confidence interval. ^1^ For black-coffee consumers, non-coffee consumers and sugared-coffee consumers combined were regarded as reference group; ^2^ the ORs (95% CIs) were adjusted for age (years, continuous), sex (for men and women combined), body mass index (BMI, <23, 23 to <25, 25 to <30 and ≥30 kg/m^2^), smoking status (never smokers, ≤10 and >10 pack-years), alcohol consumption (non-drinkers, ≤5, 5.1 to ≤10 and >10 g/day), family history of type 2 diabetes (yes or no), total energy intake (kcal/day, continuous), and the amount of sugar added in coffee (0, ≤5, 5.1 to ≤10, 10.1 to ≤15 and >15 g/day).

**Table 4:** Multivariate-adjusted ORs and 95% CIs for the risk of prediabetes and type 2 diabetes combined according to sugared coffee consumption.

	Non-Sugared-Coffee Consumers ^1^	Sugared-Coffee Consumers			*p* for Trend
		<1 Cup/Day	1 to <2 Cups/Day	≥2 Cups/Day	
Men and women combined					0.005
Case/total	834/1139	644/892	749/986	721/1037	
ORs (95% CIs) ^2^	Reference	0.84 (0.68, 1.03)	1.11 (0.90, 1.35)	0.73 (0.60, 0.89)	
Men					0.015
Case/total	310/399	335/442	357/425	465/638	
ORs (95% CIs) ^2^	Reference	0.83 (0.59, 1.15)	1.45 (1.01, 2.08)	0.71 (0.52, 0.97)	
Women					0.080
Case/total	524/740	309/450	392/561	256/399	
ORs (95% CIs) ^2^	Reference	0.84 (0.64, 1.09)	0.96 (0.75, 1.23)	0.75 (0.57, 0.99)	

Abbreviations: OR, odds ratio; 95% CI, 95% confidence interval. ^1^ For sugared-coffee consumers, non-coffee consumers and black-coffee consumers combined were regarded as reference group; ^2^ the ORs (95% CIs) were adjusted for age (years, continuous), sex (for men and women combined), body mass index (BMI, <23, 23 to <25, 25 to <30 and ≥30 kg/m^2^), smoking status (never smokers, ≤10, 10.1 to ≤20, 20.1 to ≤30 and >30 pack-years), alcohol consumption (non-drinkers, ≤5, 5.1 to ≤10, 10.1 to ≤20 and >20 g/day), family history of type 2 diabetes (yes or no), and total energy intake (kcal/day, continuous).

**Table 5:** Multivariate-adjusted ORs and 95% CIs for the risk of prediabetes and type 2 diabetes combined by genetic risk scores according to black coffee consumption.

Genetic Risk Scores ^1^	Non-Black-Coffee Consumers ^2^	Black-Coffee Consumers			*p* for Interaction
		<1 Cup/Day	1 to <2 Cups/Day	≥2 Cups/Day	
0 point					0.261
Case/total	1639/2204	47/61	25/39	46/61	
ORs (95% CIs) ^3^	Reference	1.03 (0.54, 1.97)	0.67 (0.33, 1.35)	0.87 (0.46, 1.66)	
0.1 to <5 points					
Case/total	524/721	12/18	8/11	9/17	
ORs (95% CIs) ^3^	Reference	1.00 (0.33, 3.06)	0.90 (0.21, 3.94)	0.49 (0.17, 1.44)	
5 to 10 points					
Case/total	596/854	14/21	14/20	14/27	
ORs (95% CIs) ^3^	Reference	0.78 (0.28, 2.15)	0.89 (0.31, 2.58)	0.36 (0.15, 0.88)	

Abbreviations: OR, odds ratio; 95% CI, 95% confidence interval. ^1^ Genetic risk scores were calculated by 5 SNPs related to coffee consumption weighted by relative effect size (β coefficient); ^2^ for black-coffee consumers, non-coffee consumers and sugared-coffee consumers combined were regarded as reference group; ^3^ the ORs (95% CIs) were adjusted for age (years, continuous), sex (for men and women combined), body mass index (BMI, <23, 23 to <25, 25 to <30 and ≥30 kg/m^2^), smoking status (never smokers, ≤10 and >10 pack-years), alcohol consumption (non-drinkers, ≤5, 5.1 to ≤10 and >10 g/day), family history of type 2 diabetes (yes or no), total energy intake (kcal/day, continuous), and the amount of sugar added in coffee (0, ≤5, 5.1 to ≤10, 10.1 to ≤15 and >15 g/day).

**Table 6:** Multivariate-adjusted ORs and 95% CIs for the risk of prediabetes and type 2 diabetes combined by genetic risk scores according to sugared coffee consumption.

Genetic Risk Scores ^1^	Non-Sugared-Coffee Consumers ^2^	Sugared-Coffee Consumers			*p* for Interaction
		<1 Cup/Day	1 to <2 Cups/Day	≥2 Cups/Day	
0 point					0.608
Case/total	521/698	397/544	442/571	397/552	
ORs (95% CIs) ^3^	Reference	0.79 (0.61, 1.04)	1.11 (0.85, 1.46)	0.74 (0.56, 0.97)	
0.1 to <5 points					
Case/total	139/196	121/162	151/196	142/213	
ORs (95% CIs) ^3^	Reference	1.02 (0.62, 1.67)	1.28 (0.79, 2.07)	0.71 (0.45, 1.12)	
5 to 10 points					
Case/total	174/245	126/186	156/219	182/272	
ORs (95% CIs) ^3^	Reference	0.79 (0.51, 1.22)	1.03 (0.68, 1.57)	0.74 (0.49, 1.12)	

Abbreviations: OR, odds ratio; 95% CI, 95% confidence interval. ^1^ Genetic risk scores were calculated by 5 SNPs related to coffee consumption weighted by relative effect size (β coefficient); ^2^ for sugared-coffee consumers, non-coffee consumers and black-coffee consumers combined were regarded as reference group; ^3^ the ORs (95% CIs) were adjusted for age (years, continuous), sex (for men and women combined), body mass index (BMI, <23, 23 to <25, 25 to <30 and ≥30 kg/m^2^), smoking status (never smokers, ≤10, 10.1 to ≤20, 20.1 to ≤30 and >30 pack-years), alcohol consumption (non-drinkers, ≤5, 5.1 to ≤10, 10.1 to ≤20 and >20 g/day), family history of type 2 diabetes (yes or no), and total energy intake (kcal/day, continuous).

**Table 7:** Multivariate-adjusted ORs and 95% CIs for the risk of prediabetes and type 2 diabetes combined by 5 coffee-related SNPs according to black coffee consumption.

	Non-Black-Coffee Consumers ^1^	Black-Coffee Consumers			*p* for Interaction
		<1 Cup/Day	1 to <2 Cups/Day	≥2 Cups/Day	
rs2074356					0.171
GG					
Case/total	1989/2694	54/71	33/48	52/73	
ORs (95% CIs) ^2^	Reference	1.07 (0.59, 1.94)	0.83 (0.43, 1.61)	0.77 (0.44, 1.35)	
GA+AA					
Case/total	770/1085	19/29	14/22	17/32	
ORs (95% CIs) ^2^	Reference	0.72 (0.31, 1.71)	0.65 (0.25, 1.70)	0.37 (0.16, 0.84)	
rs11066015					0.143
GG					
Case/total	1894/2557	54/69	29/43	51/70	
ORs (95% CIs) ^2^	Reference	1.24 (0.67, 2.30)	0.81 (0.41, 1.62)	0.84 (0.47, 1.51)	
GA+AA					
Case/total	865/1222	19/31	18/27	18/35	
ORs (95% CIs) ^2^	Reference	0.56 (0.25, 1.27)	0.68 (0.28, 1.65)	0.35 (0.16, 0.75)	
rs12229654					0.366
TT					
Case/total	2049/2765	55/73	31/47	52/72	
ORs (95% CIs) ^2^	Reference	1.02 (0.57, 1.83)	0.69 (0.36, 1.32)	0.75 (0.42, 1.33)	
TG+GG					
Case/total	710/1014	18/27	16/23	17/33	
ORs (95% CIs) ^2^	Reference	0.82 (0.33, 2.01)	0.94 (0.35, 2.52)	0.42 (0.19, 0.93)	
rs11065828					0.460
CC					
Case/total	1909/2575	51/70	26/42	52/71	
ORs (95% CIs) ^2^	Reference	0.88 (0.49, 1.56)	0.60 (0.31, 1.18)	0.81 (0.45, 1.45)	
CA+AA					
Case/total	850/1204	22/30	21/28	17/34	
ORs (95% CIs) ^2^	Reference	1.17 (0.48, 2.87)	1.19 (0.46, 3.06)	0.36 (0.17, 0.80)	
rs79105258					0.395
CC					
Case/total	1986/2672	52/70	31/48	51/70	
ORs (95% CIs) ^2^	Reference	0.98 (0.55, 1.77)	0.66 (0.35, 1.25)	0.79 (0.44, 1.42)	
CA+AA					
Case/total	773/1107	21/30	16/22	18/35	
ORs (95% CIs) ^2^	Reference	0.91 (0.38, 2.17)	1.04 (0.37, 2.92)	0.40 (0.18, 0.87)	

Abbreviations: OR, odds ratio; 95% CI, 95% confidence interval. ^1^ For black-coffee consumers, non-coffee consumers and sugared-coffee consumers combined were regarded as reference group; ^2^ the ORs (95% CIs) were adjusted for age (years, continuous), sex (for men and women combined), body mass index (BMI, <23, 23 to <25, 25 to <30 and ≥30 kg/m^2^), smoking status (never smokers, ≤10 and >10 pack-years), alcohol consumption (non-drinkers, ≤5, 5.1 to ≤10 and >10 g/day), family history of type 2 diabetes (yes or no), total energy intake (kcal/day, continuous), and the amount of sugar added in coffee (0, ≤5, 5.1 to ≤10, 10.1 to ≤15 and >15 g/day.

## Supplementary Materials

The following are available online at https://www.mdpi.com/2072-6643/12/8/2228/s1. Table S1: Baseline characteristics of the participants included in the GWAS on coffee consumption; Table S2: The significant SNPs discovered from the GWAS on coffee consumption; Table S3: The significant SNPs discovered from the GWAS on coffee consumption after additionally adjusted for BMI.
