# Supplementary material for: Interactions of Habitual Coffee Consumption by Genetic Polymorphisms with the Risk of Prediabetes and Type 2 Diabetes Combined
Source: Nutrients. 2020 Jul 26;12(8):2228. doi: 10.3390/nu12082228 (PMC7468962; doi:10.3390/nu12082228)
Supplement: Supplementary file 1 [file nutrients-12-02228-s001.pdf]

Table S1. Baseline characteristics of the participants included in the GWAS on coffee consumption

	**Non-coffee consumers (n=1,788)**	**Coffee consumers (n=6,080)**
Age, mean ± SD (years)	54.7 ± 9.0	50.8 ± 8.6
Sex, n (%)		
Men	645 (36.1)	3,040 (50.0)
Women	1,143 (64.9)	3,040 (50.0)
BMI, mean ± SD (kg/m²)	24.1 ± 3.2	24.7 ± 3.1
Alcohol consumption, n (%)		
Never drinkers	1,184 (66.2)	2,949 (48.5)
≤5 g/day	215 (12.0)	1,112 (18.3)
5 to ≤10 g/day	73 (4.1)	462 (7.6)
10 to ≤20 g/day	108 (6.1)	554 (9.1)
>20 g/day	208 (11.6)	1,003 (16.5)

Abbreviations: SD, standard deviation; BMI, body mass index.

Table S2. The significant SNPs discovered from the GWAS on coffee consumption

Chr	SNP	Position	Locus	Minor allele	Major allele	OR [1]	Beta [2]	*P*-value [3]
12	rs2074356	112,645,401	12q24.13	A	G	1.375	0.3185	6.62×10^{-8}
12	rs116873087	112,511,913	12q24.13	C	G	1.343	0.2949	1.68×10^{-7}
12	rs144504271	112,627,350	12q24.13	A	G	1.352	0.3016	1.74×10^{-7}
12	rs3782886	112,110,489	12q24.12	C	T	1.330	0.2852	2.67×10^{-7}
12	rs11066001	112,119,171	12q24.12	C	T	1.330	0.2852	3.75×10^{-7}
12	rs79105258	111,718,231	12q24.11	A	C	1.338	0.2912	3.89×10^{-7}
12	rs4646776	112,230,019	12q24.12	C	G	1.322	0.2791	4.75×10^{-7}
12	rs12231737	112,574,616	12q24.13	T	C	1.318	0.2761	5.07×10^{-7}
12	rs3809297	111,609,727	12q24.11	T	G	1.326	0.2822	5.26×10^{-7}
12	rs671	112,241,766	12q24.12	A	G	1.317	0.2754	6.67×10^{-7}
12	rs2188380	111,386,127	12q24.11	C	T	1.336	0.2897	1.21×10^{-6}
12	rs149607519	111,389,437	12q24.11	G	C	1.336	0.2897	1.21×10^{-6}
12	rs11065828	111,629,389	12q24.11	A	C	1.304	0.2654	1.26×10^{-6}
12	rs12229654	111,414,461	12q24.11	G	T	1.332	0.2867	1.49×10^{-6}
12	rs11066325	112,930,475	12q24.13	C	T	1.293	0.2570	5.13×10^{-6}
12	rs12227162	111,367,244	12q24.11	T	C	1.315	0.2738	5.51×10^{-6}
12	rs11066280	112,817,783	12q24.13	A	T	1.283	0.2492	6.02×10^{-6}
12	rs11066015	112,168,009	12q24.12	A	G	1.280	0.2469	7.79×10^{-6}

Abbreviations: Chr, chromosome; SNP, single nucleotide polymorphism; OR, odds ratio. [1] Odds ratio for the habitual coffee consumption; [2] Beta (β) coefficient was obtained from the GWAS; [3] *P*-value was calculated using a Wald test from logistic regression model adjusted for age (years; continuous), sex, and alcohol consumption (g/day; continuous).

Table S3. The significant SNPs discovered from the GWAS on coffee consumption after additionally adjusted for BMI

Chr	SNP	Position	Locus	Minor allele	Major allele	OR [1]	Beta [2]	*P*-value [3]
12	rs2074356	112,645,401	12q24.13	A	G	1.386	0.3264	3.31×10^{-8}
12	rs144504271	112,627,350	12q24.13	A	G	1.364	0.3104	8.02×10^{-8}
12	rs116873087	112,511,913	12q24.13	C	G	1.351	0.3008	1.06×10^{-7}
12	rs3782886	112,110,489	12q24.12	C	T	1.341	0.2934	1.37×10^{-7}
12	rs11066001	112,119,171	12q24.12	C	T	1.341	0.2934	1.94×10^{-7}
12	rs79105258	111,718,231	12q24.11	A	C	1.346	0.2971	2.44×10^{-7}
12	rs12231737	112,574,616	12q24.13	T	C	1.326	0.2822	3.07×10^{-7}
12	rs4646776	112,230,019	12q24.12	C	G	1.329	0.2844	3.09×10^{-7}
12	rs3809297	111,609,727	12q24.11	T	G	1.333	0.2874	3.41×10^{-7}
12	rs671	112,241,766	12q24.12	A	G	1.324	0.2807	4.33×10^{-7}
12	rs2188380	111,386,127	12q24.11	C	T	1.348	0.2986	6.10×10^{-7}
12	rs149607519	111,389,437	12q24.11	G	C	1.348	0.2986	6.10×10^{-7}
12	rs12229654	111,414,461	12q24.11	G	T	1.343	0.2949	8.10×10^{-7}
12	rs11065828	111,629,389	12q24.11	A	C	1.306	0.2670	1.16×10^{-6}
12	rs11066325	112,930,475	12q24.13	C	T	1.304	0.2654	2.77×10^{-6}
12	rs12227162	111,367,244	12q24.11	T	C	1.326	0.2822	2.93×10^{-6}
12	rs11066280	112,817,783	12q24.13	A	T	1.291	0.2554	3.85×10^{-6}
12	rs11066015	112,168,009	12q24.12	A	G	1.285	0.2508	5.70×10^{-6}

Abbreviations: Chr, chromosome; SNP, single nucleotide polymorphism; OR, odds ratio. [1] Odds ratio for the habitual coffee consumption; [2] Beta (β) coefficient was obtained from the GWAS; [3] *P*-value was calculated using a Wald test from logistic regression model adjusted for age (years; continuous), sex, alcohol consumption (g/day; continuous), and body mass index (BMI, <23, 23 to <25, 25 to <30 and ≥30 kg/m²).
